# ST‐Elevation Myocardial Infarction Secondary to Intracoronary Air Embolism During Atrial Fibrillation Ablation: Case Report and Successful Management

**DOI:** 10.1155/cric/4448297

**Published:** 2026-02-04

**Authors:** Eduardo I. Arteaga-Chan, Luis R. Cano-del Val Meraz, Rafael A. Sandoval-Espadas, Carlos A. Castro-Garcia, Andres Aguilar-Silva, Fernando Huerta-Liceaga

**Affiliations:** ^1^ Department of Interventional Cardiology, Hospital Central Sur de Alta Especialidad PEMEX, Mexico City, Mexico; ^2^ Department of Cardiac Electrophysiology, Hospital Central Sur de Alta Especialidad PEMEX, Mexico City, Mexico

## Abstract

We present the case of a 47‐year‐old woman with persistent atrial fibrillation who underwent catheter ablation. During the procedure, real‐time ST‐segment elevation was observed on surface ECG. An emergency coronary angiography revealed an air embolism in the proximal segment of the left anterior descending (LAD) coronary artery. Intracoronary adenosine was administered, successfully restoring coronary flow (TIMI 3). The procedure was aborted, and the patient was subsequently managed in the coronary care unit due to biventricular cardiogenic shock secondary to myocardial stunning. This case highlights the importance of strict hemodynamic monitoring and prompt recognition and response to uncommon complications during catheter ablation procedures.

## 1. Introduction

Catheter ablation is a widely used and effective treatment for drug‐refractory atrial fibrillation [1]. While major complications are uncommon, rare events such as coronary air embolism can lead to profound hemodynamic collapse and become life‐threatening if not promptly recognized and managed. The reported incidence of symptomatic coronary air embolism during cardiac procedures is low, estimated at 0.1%–0.3%, but its clinical impact is disproportionately severe [2]. Key procedural risk factors include the use of long sheaths in a semi‐upright position, inadequate flushing of catheters and sheaths, and complex manipulations involving open‐irrigation systems within the left atrium [1, 3, 4]. We report a case of left anterior descending (LAD) coronary air embolism complicated by cardiogenic shock and concurrent cerebral air embolism during pulmonary vein isolation. This case underscores the critical importance of preventive strategies, immediate recognition based on characteristic signs and the role of a structured, multidisciplinary management approach to ensure a successful outcome.

## 2. Case Presentation

A 47‐year‐old woman with persistent atrial fibrillation and no major cardiovascular history was scheduled for point‐by‐point pulmonary vein isolation under general anesthesia. Left‐sided ablation was completed, and right‐sided access was initiated. During the procedure, the surface ECG showed ST‐segment elevation in precordial leads (V1–V6), as well as in Leads II, III, and aVF, with progressive real‐time ST elevation and subsequently right bundle branch block with anterior and inferior ST‐segment elevation (Figures 1a, 1b, and 1c).

Figure 1(a–c) Live evolution of ST‐segment elevation during the catheter ablation procedure. (a) Top left: sinus rhythm tracing (Minute 0). (b) Top right: right bundle branch block with ST elevation in the anterior and inferior aspect (Minute 2). (c) Bottom center: ST elevation in the anterior and inferior aspect (Minute 4).

**Figure:**
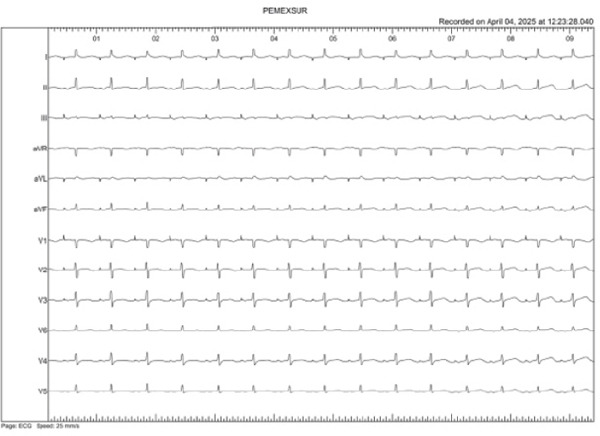
(a)

**Figure:**
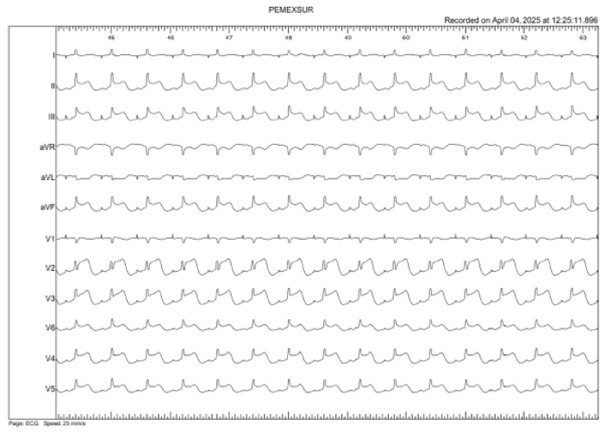
(b)

**Figure:**
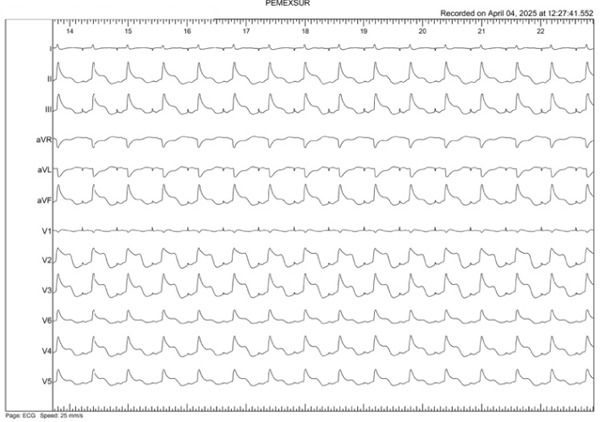
(c)

The patient developed hypotension and tachycardia (130 bpm), indicating hypoperfusion. An urgent cardiology consult was obtained, and an emergency coronary angiography was performed via right femoral artery access using a 6 Fr introducer and a 5 Fr TIG 3.5 diagnostic catheter. No coronary flow (TIMI 0) was observed in the mid–LAD artery, consistent with an air embolism. A 6 Fr JL 3.5 guiding catheter was used to cannulate the left main coronary artery followed by mechanical flushing with saline to ensure complete de‐airing. A 0.014^”^ × 180 cm Runthrough NS floppy intracoronary guidewire was advanced into the LAD, and 400 mcg of intracoronary adenosine was administered, restoring TIMI 3 coronary flow (Figures 2a, 2b, 2c, and 2d).

Figure 2(a–d) (a,b) Top left and right: During angiography, a complete occlusion of the left anterior descending coronary artery was found. (c) Bottom left: A 0.014^”^ × 180 cm Runthrough NS floppy intracoronary guidewire was advanced into the LAD and intracoronary adenosine was administered, and the coronary embolism was resolved. (d) Bottom right: Control angiography showed recovery of flow in the left anterior.

**Figure:**
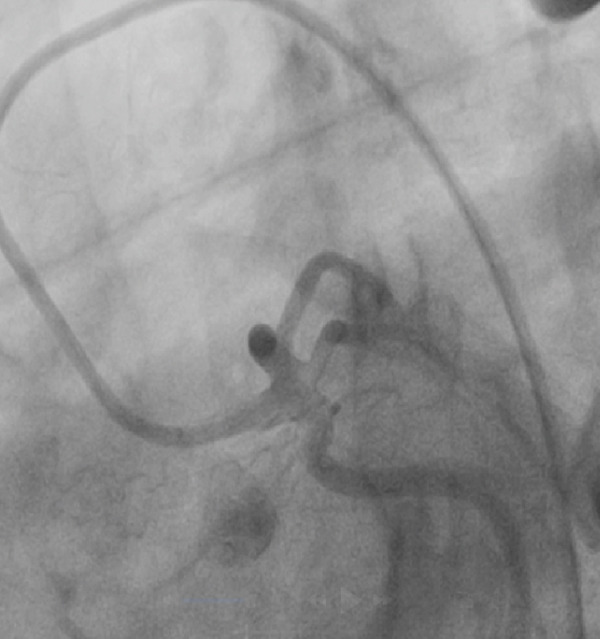
(a)

**Figure:**
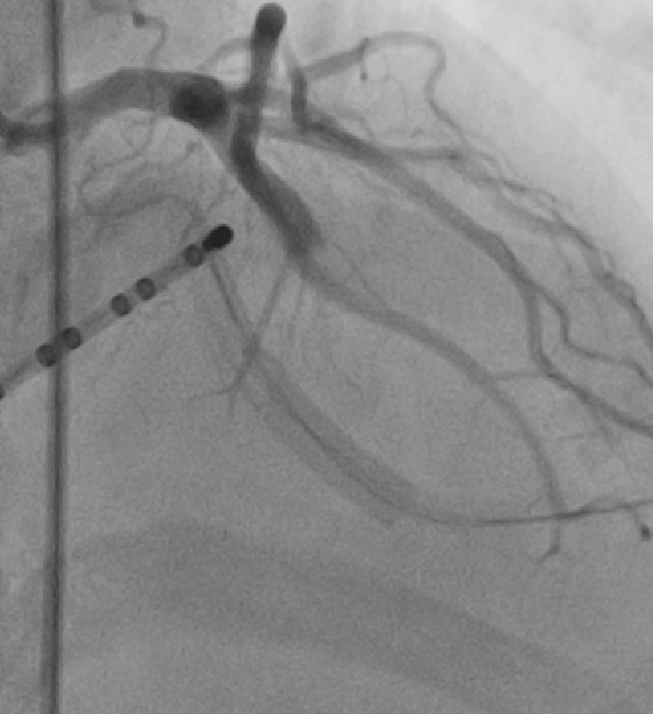
(b)

**Figure:**
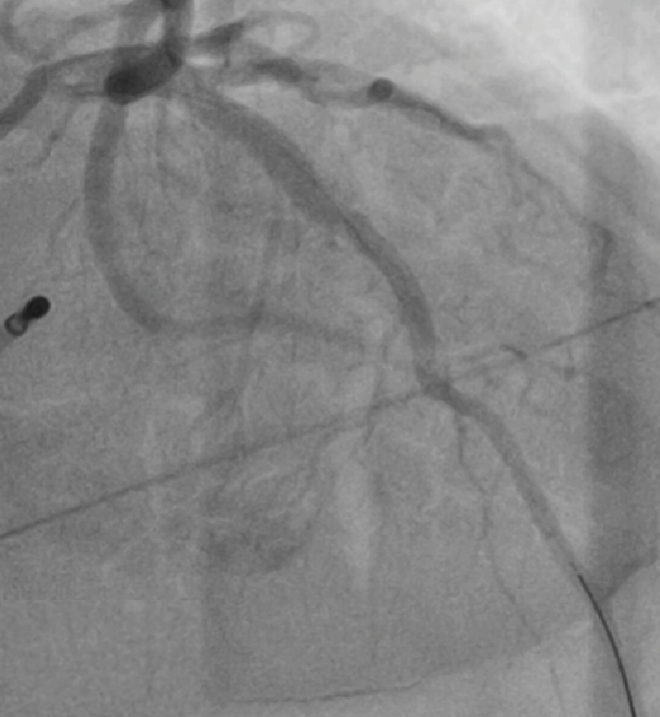
(c)

**Figure:**
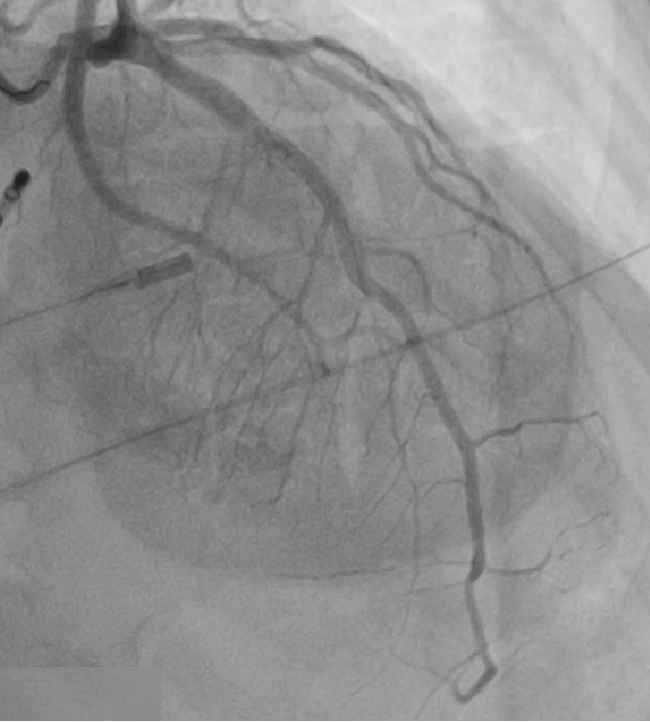
(d)

Due to this complication, the procedure was discontinued before completing right pulmonary vein ablation. A subsequent echocardiogram revealed a global right ventricular hypokinesia with anterolateral and inferoseptal wall motion abnormalities. The patient was transferred to the coronary care unit, where she developed biventricular cardiogenic shock (SCAI Stage E) due to severe myocardial stunning. Management included high‐dose vasopressors and inotropes, intra‐aortic balloon pump support, and venoarterial ECMO due to ongoing signs of tissue hypoperfusion.

At 24 h, repeat coronary angiography ruled out recurrent embolic events or underlying coronary lesions. After 48 h of circulatory support, native cardiac output improved, allowing successful ECMO and balloon pump weaning without complications. The patient was discharged 1 week after the index event. However, she developed right hemifacial and hemibody hemiplegia. A cranial CT scan revealed a left parietal cerebrovascular disease secondary to air embolism.

## 3. Discussion

Coronary air embolism is a rare but potentially catastrophic complication most associated with invasive cardiovascular procedures, including cardiac surgery, catheter‐based interventions, and percutaneous transthoracic lung biopsy [2, 3, 5].

The pathophysiology typically involves procedural events that allow inadvertent introduction of air into the coronary circulation [1, 2, 4]. As illustrated in our case, specific risk factors during ablation procedures are paramount and include:

1. Inadequate flushing: Failure to meticulously flush sheaths, catheters, and all connection tubing before insertion or connection [1, 3].
2. Sheath and catheter management: The use of long sheaths, multiple‐port manifolds, and syringe or tubing changes without proper clamping or purging [2, 3].
3. Pressure dynamics: Negative intracardiac pressure, particularly in the left atrium, which can actively entrain air through small openings [1, 4].
4. Procedural setup: Performing the procedure with the patient in a semi‐upright position and desynchronization of open‐irrigation systems, which can introduce air into the circulation [1].

Clinical outcomes are highly variable and depend on the volume and location of air embolism. Acute presentations may include ST‐elevation myocardial infarction, hemodynamic instability, arrhythmias, heart block, and cardiac arrest, with the right coronary artery territory most frequently affected due to its anatomical position in supine patients [1, 2]. Neurological sequelae and death occur in up to one‐third of symptomatic cases, particularly when emboli reach the coronary or intracranial circulation [2]. Even small volumes of air can cause significant morbidity due to microvascular obstruction, endothelial injury, and thromboinflammatory responses [4, 5]. Diagnosis is confirmed angiographically by the presence of radiolucent, mobile filling defects within the coronary artery. Our case, featuring abrupt ST‐elevation and subsequent cardiogenic shock, is consistent with this classic presentation.

A review of the literature reveals a spectrum of outcomes. Many small, distal emboli resolve spontaneously. The cornerstone of treatment is the administration of 100% supplemental oxygen to facilitate nitrogen washout and accelerate air bubble resorption [6, 7]. Direct aspiration of the air bubble through the angiographic catheter is highly effective and should be attempted immediately [6]. In cases of refractory microvascular dysfunction, intracoronary vasodilators may be beneficial. Supportive care, including fluid resuscitation and inotropic support, is crucial for hemodynamic instability [6, 7].

Hyperbaric oxygen therapy is recommended for severe cases, especially those with neurological or persistent cardiac dysfunction, as it accelerates air resorption and mitigates ischemic injury [6, 7]. However, as in our case, large‐volume emboli in a proximal vessel like the LAD can have more severe consequences. Compared with previously reported cases, our patient′s course was notable for the progression to profound myocardial stunning and cardiogenic shock requiring mechanical circulatory support, despite successful air dispersal and the absence of underlying coronary disease. This highlights that the primary injury is not just vascular occlusion but also intense microvascular spasm and dysfunction [4, 5]. Furthermore, the concomitant focal neurological deficit is a rare but documented finding, signifying a paradoxical cerebral air embolism and underscoring the systemic potential of this complication. The successful use of mechanical circulatory support, as demonstrated here, can be a lifesaving bridge to recovery in cases of severe myocardial stunning.

Early identification is essential to prevent severe outcomes. In this case, ST‐segment changes, and signs of hypoperfusion prompted immediate diagnosis and effective intervention. Coronary angiography confirmed an air embolism in the proximal LAD, and intracoronary adenosine administration resulted in reperfusion and clinical improvement.

This case is notable for the evolution into cardiogenic shock and focal neurological deficits consistent with cerebral air embolism. The successful use of mechanical circulatory support, as demonstrated here, can be a lifesaving bridge to recovery in cases of severe myocardial stunning.

Desynchronization of irrigation systems—such as those used in radiofrequency ablation—also predispose to inadvertent air entry. Multidisciplinary coordination was essential for the successful outcome in this case.

Preventive measures are procedural and technical: meticulous de‐airing during cardiac surgery, continuous venting of air from the heart until bubbles are sparse and nonbuoyant, use of transesophageal echocardiography for real‐time detection, and effective collaboration among surgical, anesthesia, and perfusion teams [4]. During percutaneous procedures, minimizing needle size, avoiding aspiration biopsy when possible, and careful catheter management are essential [2, 3]. Awareness of risk factors and adherence to evidence‐based protocols are paramount in reducing incidence [2–4].

## 4. Conclusion

Although rare, coronary air embolism during atrial fibrillation ablation is a potentially lethal complication. Prompt recognition and immediate management can prevent adverse outcomes. This case underscores the importance of continuous electrocardiographic monitoring, the immediate availability of interventional cardiology and cardiovascular anesthesia teams during complex ablation procedures. The successful use of mechanical circulatory support as bridge to recovery in cases of severe myocardial stunning.

## Consent

No written consent has been obtained from the patient as there is no patient identifiable information included in this case report.

## Conflicts of Interest

The authors declare no conflict of interest.

## Funding

No funding was received for this manuscript.

## Data Availability

Data sharing not applicable to this article as no datasets were generated or analyzed during the current study.
